# The Effect of Dental Implant Drills Materials on Heat Generation in Osteotomy Sites: A Systematic Review

**DOI:** 10.1055/s-0043-1768472

**Published:** 2023-06-19

**Authors:** Shreyasi Chakraborty, Mohammad-Adel Moufti, Waad Kheder

**Affiliations:** 1 Department of Implant Dentistry, BPP University, Birmingham, United Kingdom; 2 Department of Preventive and Restorative Dentistry, College of Dental Medicine University of Sharjah, Sharjah, United Arab Emirates; 3 Research Institute for Medical and Health Sciences, University of Sharjah, Sharjah, United Arab Emirates

**Keywords:** ceramics, dental implant, drills, friction, heat, osteotomy

## Abstract

The aim of this review was to examine the impact of dental implant drill materials and wear profiles on heat generation in the osteotomy sites as reported in experimental studies and to critically appraise these studies. The research question was formulated based on predefined patient, intervention, comparison, and outcome (PICO) elements. A comprehensive electronic search was undertaken in Medline/PubMed Central, Science Direct, and Google Scholar, using predetermined keywords, followed by a manual search of the bibliography of the selected articles. The selection of the studies for the critical appraisal part of our study was based on the criteria used to assess the study designs such as study aims, outcome measure, clarity of method, sample selection, randomization, allocation concealment, sample attrition, bias, method of data analysis, and external validity. Increased heat generation was observed with both ceramic and metal drills; the heat generation was proportional to drills' wear. The literature was inconclusive regarding the association between drill material and heat generation. However, drill materials had a significant influence on the overall temperature increase during osteotomy. The noncoated drills showed a higher wear resistance, and it has been observed that using worn drills leads to more friction contact, reduced drill cutting efficiency, and increased heat generation. Eleven *in vitro* studies met the inclusion criteria, and showed a considerable methodological heterogeneity and confounding factors, including drill geometry, speed and load, depth and diameter, number of uses, irrigation protocol, study specimens, and the heat measuring device. Besides, most of the studies have a potential operator and assessor bias, and some have sponsorship bias. It is possible to conclude that the literature is not conclusive on the effect of drill materials on heat generation during osteotomy. Lack of standardization and uniformity in the study design, along with potential bias in the study methodology can be the reason for the heterogeneity of the results.

## Introduction

One of the most important challenges in bone drilling during osteotomy site preparation is heat generation and thermal damages to the alveolar bone. This could be due to several biomechanical factors such as the drilling protocol (technique, force, speed, drill geometry, and irrigation), 1 properties of the drills, 2 and properties of the bone. 3 4 Investigations have reported that temperatures of over 44°C lead to irreversible damage. Drill wear is one of the important causes of the rise in temperature during osteotomy, which needs to be controlled to avoid thermal osteonecrosis. 5 The elevated bone temperature constitutes a major cause of early dental implant failure since it may lead to hyperemia, osteocyte degeneration, increased osteoclastic activity, and necrosis, which ultimately affects the regenerative capacity of the bone and consequently ends with osseointegration failure. 4 Delayed healing, failure of osseointegration, and necrosis of the bone were reported during the osteotomy when the temperature exceeded 47°C for more than 1 minute. 6 Therefore, for successful osseointegration, the osteotomy preparation in low temperature is of utmost importance.

For many decades, dental implant drills were made of stainless steel because of their high cutting efficiency and durability. Ceramic drills, which are mainly composed of 80% zirconia oxide and 20% alumina oxide, were lately introduced in the market. Combining zirconia with magnesium or alumina stabilizes zirconia, resulting in better biomechanical properties. 7 8 Ceramic drills have the advantage of being biocompatible, which reduces the chances of allergic responses to metal during osteotomy preparation. They show good resistance against high temperature, corrosion, and wear/abrasion. 9 Thus, they are expected to generate less heat during implant site preparation. 10 However, literature on the use of ceramic drills in implantology is scarce and is not conclusive on their advantages in lowering heat generation during drilling. Understanding whether drill material can affect heat generation during osteotomy preparation is of high clinical relevance as it can influence the implant success and survival rate.

The effect of the drill material on heat generation during osteotomy has been debated. Although some reports confirmed this, other research claims that the tool material does not have a significant effect on heat generation during osteotomy. 11 Despite the advances in the manufacturing of ceramic burs, only a limited number of studies have tried to address such an important effect, and there is still a lack of consensus when it comes to its dental applications. 12 The aim of this study was to explore the experimental studies in the literature regarding the impact of drill materials and their wear profile on heat generation in osteotomy sites, and to critically appraise these studies.

## Methods

### Articles Search Strategy

**Fig. 1:**
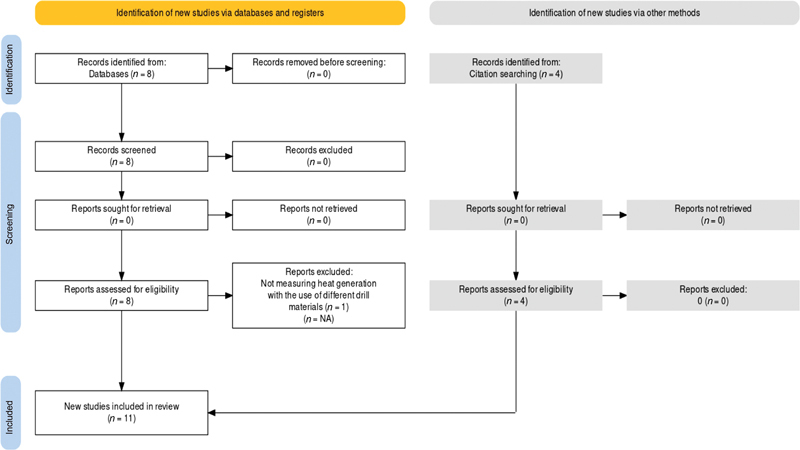
Flowchart of the articles search strategy.

The research question “Is there a difference in the amount of heat generation using different drill materials for osteotomy preparation?” was formulated based on predefined patient, intervention, comparison, and outcome (PICO) elements that are explained below. The search was undertaken in Medline/PubMed Central, Science Direct, and Google Scholar using the following keywords and Boolean operators: “implant site preparation” OR “osteotomy” OR “bone drill*” AND “ceramic drill” OR “zirconia drill” OR “zirconium drill” OR “zirconia oxide drill” OR “Zircon* based drill” OR “Diamond Like Carbon coated drill” OR “DLC coated drills” OR “black diamond drill” OR “titanium nitride coated metal drills” OR “tungsten carbide coated metal drill” OR “tin coated drill” OR “stainless steel drill” OR “stainless steel drill” OR “titanium drill” AND “heat generation” OR “thermal change” OR “thermal osteonecrosis” OR “thermal variation” OR “intrabony temperature” OR “temperature change.” The selection of articles/studies followed the standardized staged process of the Preferred Reporting Items for Systematic Reviews and Meta-Analyses (PRISMA) as illustrated in Fig. 1 . First, the abstracts of the initially selected articles were screened by the authors SC and WK independently based on the inclusion and exclusion criteria listed below to confirm the articles' relevance to our study. Any disagreement was discussed among the three authors and resolved. The full text of the selected articles was obtained and read in full, also by both reviewers SC and WK independently, to confirm the selection of those articles. The bibliographies of the studies were manually searched to identify any article that was not captured by the electronic search. Four articles were found relevant and selected for this review.

#### Inclusion Criteria

Included in this review were studies published on the influence of drill material on heat generation in osteotomy sites, studies that answer the research question, and studies including human, animal, or synthetic bone.

#### Exclusion Criteria

Case reports, case series studies, reviews, articles published earlier than the year 2000, and publications in language other than English were excluded from our review.

### Critical Appraisal

**Fig. 2:**
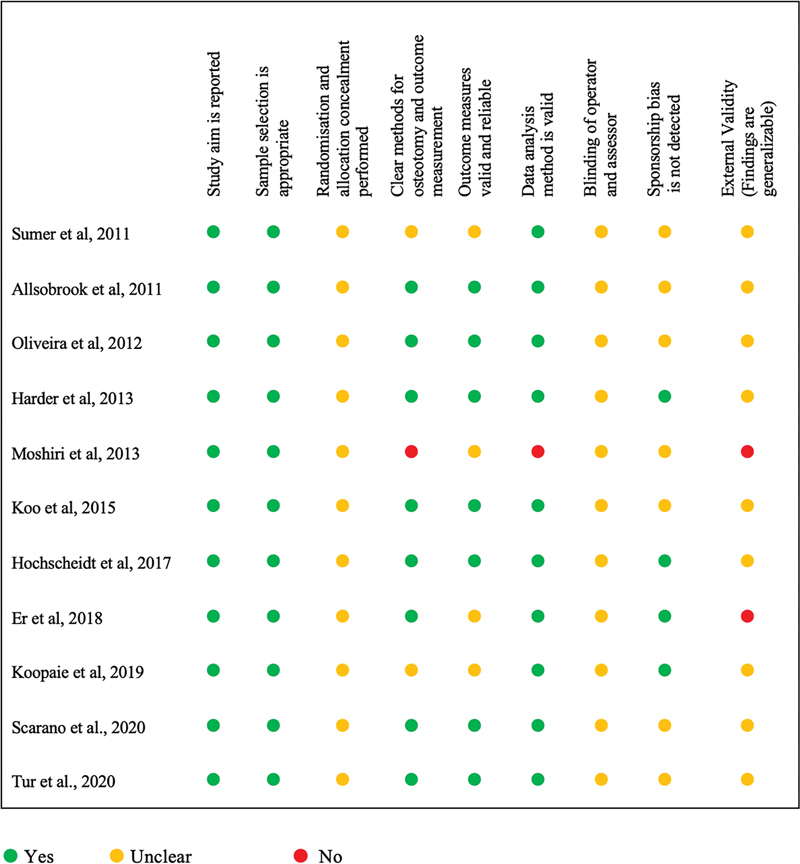
Traffic light system summarizing critical appraisal of the included articles.

Due to lack of standardized critical appraisal tools for *in vitro* studies, our study selected appraisal criteria based on the aspects that should be assessed in this type of study designs. The aspects appraised were study aims, outcome measure, method clarity, sample selection, randomization, allocation concealment, sample attrition, bias, method of data analysis, and external validity as presented in Fig. 2 .

## Results

The initial electronic search identified eight articles. One article was excluded after abstract screening because it did not report the measurement of heat generation in relation to the different drill materials ( Table 1 ). The remaining seven articles met the inclusion criteria and were selected. An additional four articles were identified from the manual search of the bibliography of the 7 selected articles, resulting in a total of 11 articles being selected for our review ( Table 2 ). The article search strategy was designed to capture articles that go back to the year 2000; however, relevant studies were found only after 2010.

**Table 1:** List of excluded articles

SN	Study	Database	Article title
1	Batista Mendes et al 10	PubMed	Influence of implant drill materials on wear, deformation, and roughness after repeated drilling and sterilization

**Table 2:** List of included articles in a chronological order

SN	Author/year	Database	Article title
1	Sumer et al/2011	PubMed	Comparison of heat generation during implant drilling using stainless steel and ceramic drills
2	Allsobrook et al/2011	Manual search	Descriptive study of the longevity of dental implant surgery drills
3	Oliveira et al/2012	PubMed	Thermal changes and drill wear in bovine bone during implant site preparation. A comparative *in vitro* study: twisted stainless steel and ceramic drills
4	Harder et al/2013	Manual search	Influence of the drill material and method of cooling on the development of intrabony temperature during preparation of the site of an implant
5	Moshiri et al/2013	Manual search	Evaluation the effect of drill type on heat generation in implant drilling site
6	Koo et al/2015	PubMed	Effects of implant drill wear, irrigation, and drill materials on heat generation in osteotomy sites
7	Hochscheidt et al/2017	Manual search	Thermal variation during osteotomy with different dental implant drills: a standardized study in bovine ribs
8	Er et al/2018	PubMed	Improved dental implant drill durability and performance using heat and wear resistant protective coatings
9	Koopaie et al/2019	Science Direct	Comparison of wear and temperature of zirconia and tungsten carbide tools in drilling bone: *in vitro* and finite element analysis
10	Scarano et al/2020	PubMed	Infrared thermographic evaluation of temperature modifications induced during implant site preparation with steel vs. zirconia implant drill
11	Tur et al/2020	PubMed	Thermal effects of various drill materials during implant site preparation—ceramic vs. stainless steel drills: a comparative *in vitro* study in a standardised bovine bone model

All the included articles were *in vitro* studies, of which 10 were performed on bovine bone specimens except 1 study that was performed on artificially manufactured bone specimens. Drills used in the studies were fabricated from different materials such as stainless steel, ceramic, tungsten carbide, black diamond, and diamond like carbon-coated drills. Ten of the studies used thermocouples for temperature measurement placed close to the osteotomy at different depths to measure the real-time temperature. In study number 11, the temperature was measured using an infrared thermometer. The studies evaluated other variables such as drill wear, effect of depth, and irrigation. The rotational speed and load used for osteotomy preparation was constant during the experiments and was reported in all studies. All selected articles observed an increase in the bone temperature while preparing the osteotomy sites. The overall temperature increase varied distinctly among the studies, which could be explained by the confounding factors in each study ( Table 3 ). Most studies reported the bone baseline temperature, while a few studies did not report the bone baseline temperature and only compared the maximum temperature reached by the different drill types. 13 14 15 16

**Table 3:** Overall temperature changes during osteotomy

Author/Year	Results
Sumer et al 9	The mean temperature in the water bath was 30.1°C. The mean maximum temperature with stainless steel drills was 35.05°C, whereas that with ceramic drills was 35.9°C
Allsobrook et al 13	The mean temperature measured over the experiment was 20.0°C. The maximum temperature reached was 27.7°C, and the temperature seldom varied 2°C from the initial temperature
Oliveira et al 11	Mean baseline bone temperature for the stainless steel and ceramic drills were 21.59 ± 0.1 and 21.6 ± 0.1°C, respectively. The mean maximum increase in temperature was 1.64 ± 1.11°C
Harder et al 12	The baseline temperature was 23°C. The mean maximum intrabony temperature increased was 3.9°C
Moshiri et al 19	Baseline temperature not mentioned. The mean maximum temperature was 33°C
Koo et al 14	The mean internal temperature is 36.5°C and the surface temperature of 28°C (baseline temperature). The mean maximum temperature recorded was 64.7°C
Hochscheidt et al 21	Baseline temperature was 20.32°C and the peak temperatures reached were as follows, in sequence: steel with a diamondlike carbon coating, 67.6°C at 5-mm drilling depth; aluminum-toughened zirconium ceramic, 52.1°C at 13-mm depth; and experimentally surface-treated steel, 32.0°C at 13-mm depth
Er et al 20	The baseline temperature was 23–24°C, with the mean maximum bone temperature at 35.5°C
Koopaie et al 22	Baseline temperature was 20°C. The mean temperature was 38.3°C
Scarano et al 15	The steel drills showed a bone temperature of 42.45 ± 1.70°C, compared with the zirconia drills, which reported average values of 40.80 ± 0.85°C
Tur et al 16	The maximum mean temperature increases for 16-mm drilling without irrigation was 27.20°C

## Discussion

Osseointegration is the direct functional and structural connection between the surface of the dental implant and the living bone. Heat generation during drilling to prepare the implant site has a significant impact on the success of osseointegration, 3 because heat-induced bone injuries reduce the implant primary stability, leading to implant failure. 17 Furthermore, if the bone is exposed during osteotomy to a temperature exceeding 47°C for more than 1 minute, irreversible cellular damage will happen, and the bone will be replaced with fibrous tissue. 18 Drill materials can influence the heat generation during osteotomy. Therefore, the selection of drills is of high clinical relevance as it can help in implant osseointegration and increase their success and survival rate. These studies have been performed *in vitro* on a variety of bone models using different temperature measurement systems (various thermocouples or infrared thermography devices).

### Composition of the Drills

Most of the included studies compared stainless steel and ceramic materials along with some less common drill types, such as tungsten carbide carbon, titanium nitride, and other coated drills. In comparison between metal and ceramic drills, the studies have reported contradictory findings. Some found that stainless steel drills generate more heat than the ceramic drills 9 11 15 19 ; others showed no difference, 12 14 20 while one study reported higher mean temperature with ceramic drills than metal drills. 16

The effect of drill coating was also examined. One study by Allsobrook et al 13 found that tungsten carbide-coated stainless steel generated less heat compared with the stainless steel drills, while Hochscheidt et al reported that steel diamondlike carbon coating showed highest temperature than other drills fabricated from surface-treated steel, and aluminum-toughened zirconium ceramic. 21 A direct comparison between the study results is neither possible nor valid, as the wear resistance of pure zirconia, for instance, is different to that of zirconium oxide coating. It is apparent that drill materials had a significant influence on the overall temperature increase during osteotomy, which may be related to a higher wear resistance of the noncoated drills, as explored below.

### Drill Wear and Number of Uses

It has been observed that using worn drills leads to more friction contact, reduced drill cutting efficiency, and increased heat generation. This may increase the possibility of bone necrosis, irreversible cellular damage, and replacement of bone tissue with fat tissue. 20 Therefore, the number of drill reuse is an important factor in heat generation during osteotomy.

The number of drill uses has varied among the reviewed studies, but most of them have used the drills for 50 times, and different conclusions were reached. Oliveira et al compared scanning electron microscope (SEM) images of both stainless steel and zirconia drills before and following 50 uses, and identified different patterns of wear. 11 The ceramic drills had slightly visible alterations on one edge of the drill tip, contrasting with the stainless steel drill, which showed a higher tip wear in both edges. However, none of the drills appeared to present severe deformation or blunting after 50 uses. This is not consistent with Koopaie et al in which the overall wear of the ceramic drills after 45 uses was about half that of the tungsten carbide drills. However, the comparator in both studies was different. 22 The SEM images in the third study by Allsobrook et al 13 found that tungsten carbide drills display least corrosion and wear and lowest mean drilling temperatures after 20 osteotomies when compared with stainless steel drills. Interestingly Scarano et al 15 found that in case of using zirconia drills, the outline of the implant bed was well defined even after 120 osteotomies. Another noteworthy finding is that by Koo et al 14 who found that only the initial/pilot drills generate more heat after 50 uses.

### Drill Geometry

The drill geometry is of critical importance to heat generation, and has been investigated in many studies. Therefore, to make a valid comparison between the studies, the geometry of the drills used in the studies should be similar. Koo et al 14 used drills of different shapes, which could affect the interpretation of the data. The other studies either have used similar drills or have not reported the design of the drills used in their experiments.

### Drill Speed and Applied Load

The speed and load used during osteotomy were also not consistent among the studies. It has been established that the increase or decrease in speed and load can affect the temperature change during implant site preparation. Hence, the results obtained in different studies are not comparable. Furthermore, not all studies reported these parameters. For instance, Koopaie et al 22 and Er et al 20 did not mention the used load in the experiments, while Tur et al 16 did not mention both the speed and load.

### Depth and Diameter of the Osteotomy

The temperature was measured in the included studies at different depths and for different diameters of the final drill. Koo et al 14 suggested that the initial (pilot) drill should be changed after 50 uses, as this drill leads to more heat generation because it is the first drill to cut the bone in the osteotomy compared with the following drills, but most of the studies have measured temperature increase with the final drill as it can provide accurate temperature change because the thermocouple placement needs to be closer to the prepared osteotomy wall.

Similarly, the temperature was measured for one particular depth in all the studies instead of measuring throughout the osteotomy and the level of depth varies in each study. Again, this raises similar doubts in the validity of comparing the studies. Few of the studies have found that the superficial part of the osteotomy cavity generates more heat compared with the deeper parts. 12 15 The study by Moshiri et al 19 found that the temperature was higher at the depth of 6 mm when compared with that at 3 and 9 mm. The authors observed that this result is similar to other studies stating that there will be more heat generation during the preparation of the superficial part of the osteotomy site. The studies explained this finding by stating that friction is higher in the superficial part due to the higher content of cortical bone. This conclusion contradicts with many previous publications on the subject, which reported higher temperature while preparing the deeper parts of the osteotomies due to ineffective cooling with irrigation. These contradictory results again raise concerns regarding the validity of the study methods.

### Irrigation during Osteotomy

The use of irrigation has been proven to decrease the amount of heat generation during osteotomy. Most of the studies have used external irrigation with saline solution, but the flow rate and solution temperature are either variant or not reported, which can affect the rigor of the studies.

### Study Models/Specimens

No human-based articles were identified, which may be due to ethical reasons, since these experiments require an invasive insertion of special equipment to detect the temperature change. Most of the included articles have used bovine bone for the experiment, which has been used successfully in many studies to investigate heat generation in response to implant site preparation. However, there is no standard study model for conducting experiments on heat generation during osteotomy. 6 Thus, the studies have used different parts of bovine bone like ribs, femur, and scapular bone to conduct the experiment, which was considered to have a similar density as the human bone. However, none of the studies reported the bone density type, despite the significant importance of bone density in heat generation and thermal conductivity. The included studies have only claimed that the density is comparable to that of the human bone.

There are other concerns regarding the generalizability of results obtained from dead bone. First, in addition to bone density, thermal conductivity varies significantly between live and dead bone. This can be due to the difference in cellularity, water content, and fluid movement. 23 Second, dead bone can be fresh or frozen and thawed. It is of paramount importance to know the baseline temperature of the bone block prior to experiment to appreciate the exact increase in the temperature during drilling. Ten of the included articles have used bovine bone specimens, which were frozen and then thawed in a thermostat-controlled water bath or immersed in saline solution to minimize the thermophysical and mechanical changes of the proteins in the dead bones. This also maintained the internal temperature of the bone specimen before the start of the experiment. Only one study by Moshiri et al 19 failed to explain the procedure used to maintain the bovine bones and the baseline temperature in the experiment.

### Temperature Changes Measurement

Thermocouples can detect only spot temperatures and not the overall thermal profile. Therefore, factors like depth of recording, distance of the sensor from the osteotomy site, and material of sensor element can affect the overall results. Nine of the included articles have used thermocouples for measuring the changes in the temperature during implant bed site preparation. But their use is not uniform, especially in respect to distance of the sensor from the osteotomy, which ranges from 0.2 to 1.5 mm. Sumer et al 9 and Moshiri et al 19 have not even mentioned the distance. However, Koopaie et al 22 have used infrared thermometer for heat measurement, which is considered a better option as it provides the overall thermal profile with less degree of error. On the other hand, Scarano et al 15 used infrared thermography for evaluating the change in temperature during implant bed preparation. There is no study in the literature that compares the accuracy of heat generation using both techniques. Hence, determining a preference is still difficult. Furthermore, despite the importance of calibrating the instrument before the experiment to verify the precision of temperature recording (device reliability), only few of the reviewed studies have reported undertaking this using a proper method for calibration.

### Study Bias

Blinding the operator and assessor is difficult in these studies as the operator needs to see the drills while performing the experiment and the assessor has to measure the heat instantaneously. Having said that, these types of bias can be reduced by assigning different operators for each group along with an independent assessor. None of the studies have performed this, which makes them all prone to considerable bias. Another important source of bias observed in these studies is sponsorship bias. Some articles failed to acknowledge the source of funding and to provide a clear statement on conflict of interest.

There are many confounding factors in studies evaluating the heat generation during the osteotomy preparation. Therefore, standardization and uniformity in the study design is required to compare the results and to make a valid conclusion. The present review attempted to standardize the processes of identifying and appraising the relevant articles in the literature despite the lack of standardized appraisal tools for *in vitro* studies. Finally, a comprehensive evaluation of all the relevant confounding factors within all the studies was conducted, which helped identify the missing gap of knowledge in the literature. The results of this study can be generalized to clinical practice with caution as the included articles were *in vitro* studies, with a significant dissimilarity between live human and dead animal bone. This, besides the remarkable heterogeneity and potential bias in the study methods.

## Conclusion

It is possible to conclude that the literature about the effect of drill materials on heat generation during osteotomy is not conclusive. The lack of standardization and uniformity in the study design, along with potential bias in the study methodology, makes it challenging to draw concrete conclusions based on the findings. However, the findings of this study can still serve as a helpful guide for clinicians seeking to optimize their approach to implant procedures.

## Clinical Implications

The findings of this review emphasize the significant impact of drill material and wear behavior on heat generation during osteotomy. It is important for clinicians to be aware of these findings, as they underscore the need to carefully inspect wear patterns and consider the appropriate drill material when performing osteotomy procedures. By taking these factors into account, clinicians can help minimize the risk of excessive heat generation, which can have a negative impact on implant success rates and treatment outcomes.

## Limitations of the Study

To minimize the effect of clinical heterogeneity and confounding factors on the study outcomes, this review focused solely on benchtop research related to the subject. Therefore, the generalizability of the findings to clinical practice may be limited.
